# The effect of preconditioning with high-intensity training on tissue levels of G-CSF, its receptor and C-kit after an acute myocardial infarction in male rats

**DOI:** 10.1186/s12872-020-01380-w

**Published:** 2020-02-11

**Authors:** Reza Ghanimati, Hamid Rajabi, Fatemeh Ramezani, Maral Ramez, Mohsen Bapiran, Farinaz Nasirinezhad

**Affiliations:** 1 Department of Exercise physiology, Faculty of Physical Education and Sport Sciences, Kharazmi University, Tehran, Iran; 2 Physiology Research Center and Physiology Department, Faculty of Medicine, Iran University of Medical Sciences, Tehran, Iran

**Keywords:** Preconditioning, High-intensity interval training, Cardioprotection, Acute myocardial infarction

## Abstract

**Background:**

Exercise training is known as a practical way to increase cardioprotection against stress, and it seems that stem cell recruitment is one of its mechanisms. The purpose of the present study was to investigate the effect of preconditioning with High-intensity interval training (HIIT) on tissue levels of G-CSF, its receptor and C-Kit following acute myocardial infarction in male rats.

**Methods:**

Twenty Male Wistar rats were randomly divided into 4 groups of control, MI, HIIT, and HIIT+MI. Training groups performed 2 weeks of high intensity interval training in 4 sections. The first section consisted training in 3 days and 2 sessions in each day (4 × 2 min with 35–40 m/min and 3 × 2 min with 25–30 m/min between high intervals. The second part included 2 days of training (4 × 2 min with 40 to 45 m/min and 3 × 2 min with 28 to 32 m /min). The third part was performed in 3 days with one more repetition. The fourth section consisted 2 days of training and with one more repetition compared to section 3. For induction of myocardial infarction, subcutaneous injection of isoprenaline was used. CK, total CK, LDH, and troponin T were measured in serum and G-CSF, G-CSFR and C-Kit proteins were measured by the Western Blot method in the heart tissue.

**Results:**

The results of this study showed that enzymes of CK, total CK, LDH, troponin T had a significant increase in both MI and HIIT+MI groups compared to the other two groups (*P* < 0.001) and these indices in the MI group were significantly higher than the HIIT+MI group. Also, the results demonstrated that G-CSF, G-CSFR and C-Kit protein expression in the heart tissue significantly increased after MI. As well as, 2 weeks of HIIT training significantly increased G-CSF and C-kit in the training group compared to the control group, but the training caused that these proteins does not increase in HIIT+MI group as much as MI group.

**Conclusions:**

Along with other protective pathways, high intensity interval training can increase cardioprotection and decrease heart injuries through the increase in G-CSF, G-CSFR and C-kit level.

## Background

The heart proper function depends on the delivery of blood and oxygen by the coronary arteries. However, some factors, such as arteriosclerosis or the formation of clots in these vessels lead to an abnormal blood supply and ischemia of the heart muscle, resulting in substantial injury to this sensitive tissue. The mechanisms responsible for ischemic injury are not fully understood. However, numerous studies have shown that several factors, including the reduction of Adenosine triphosphate (ATP), production of reactive oxygen species (ROS), accumulation of hydrogen ions, the production of reactive nitrogen species (RNS), increase of intracellular calcium, calpain activity, and leukocyte activity are involved in cardiac injury [1–4].

Recent studies have shown that increase of stem cell recruitment factors (such as Granulocyte colony-stimulating factor (G-CSF), Stromal cell-derived factor (SDF), Stem cell factor (SCF), and FMS-like tyrosine kinase 3 (Flt3)), as well as increased stem cells, acts as a protective mechanism of the heart against infarction and improves the function of the left ventricle [5–7]. The results show that stem cells are as a novel method to treat heart disease [5–7].

Further studies have shown that G-CSF, SDF, SCF, and Flt3 lead to recruitment of stem cells from bone marrow to injured tissue such as the heart. G-CSF is a 25 kDa glycoprotein cytokine that is released from endothelial, macrophages, some of the immune cells (monocytes), and also most of the injured tissues of the body, including the heart, kidneys, pancreas, and even the skeletal muscle [8]. The main function of G-CSF is regulation of proliferation, division and growth of neutrophils and granulocytes in the bone marrow [9]. It seems that after acute myocardial infarction, G-CSF and other factors be able to mobilize stem cells of bone marrow and injured tissue, and regenerate the heart tissue and produce new myocytes [10].

Under normal conditions, the levels of G-CSF and stem cells in the body are negligible [11], but it has been shown that the concentration of these factors changes in various physiological (exercise induced-hypoxia and climbing altitude) and pathological conditions (coronary artery diseases, myocardial infarction, neuroinflammation in traumatic brain injury, …) [12]. De Lisio et al. (2012) reported that 8-weeks continuous endurance training increases stem cell proliferation, which attributed to the acute increase in G-CSF [13]. Baker et al. (2011) showed an increase in G-CSF of skeletal muscle after 10 weeks of endurance training and suggested that this increase is associated with increased serum cytokines [14]. Krüger et al. (2015) reported that one session of resistance training increases the level of G-CSF, which lead to the recruitment of stem cells to damaged skeletal muscle [15].

Several approaches have been identified to achieve cardioprotection against infarction. These strategies include exercise training, ischemic preconditioning, ischemic postconditioning and pharmacological interventions [16]. Regular exposure of the heart to mild IR without cell death during conditions caused by exercise training leads to cardioprotection through the preconditioning process [17, 18]. Also, many studies show cardiovascular, muscular and metabolic adaptations are highly dependent on the intensity of training and the evidence recommended that exercise training intensity is the most critical factor in cardioprotection [19, 20]. The protective role of high-intensity interval training has been accepted during acute injury than other types of training [18]. Since the effect of exercise training on increasing the levels of stem cell recruitment factors has been investigated, and it has also been shown that the levels of stem cells and stem cell recruitment factors increase during acute cardiac injuries, therefore, this research was designed to investigate the protective effect of high-intensity interval training on the axis of the stem cell recruitment factors during cardiac injury.

## Methods

### Animals and experimental design

In this study, 20 Male Wistar rats with a weight of 224.41 ± 5.1 g (8 weeks old) were purchased from the Pasteur Institute and kept at a temperature of 22 ± 2 °C, relative humidity of 50–55% and a dark-light cycle of 12:12. Animals were randomly divided into 4 groups (5 rats/group) of control, Myocardial infarcted (MI), High-intensity interval training (HIIT), High-intensity interval training+ Myocardial infarcted (HIIT+MI). Forty-eight hours after the last training session, myocardial infarction was induced by 2 times injection of 150 mg/kg isoprenaline solution (24 h interval). One week after induction of infarction, the rats were anesthetized with ketamine (100 mg / kg) and xylazine (10 mg / Kg) and after ensuring from complete and deep anesthesia, the animal’s chest was opened and blood samples were taken from the left ventricle. Finally, the animals under deep anesthesia with ketamine (100 mg / kg) and xylazine (10 mg / Kg) were sacrificed by transcardiac perfusion with PBS followed by 4% paraformaldehyde and then the hearts were removed and frozen rapidly with liquid nitrogen and stored at-80 °C. After the end of the study, the animals were delivered to the animal care center to be destroyed in accordance with the ethical principles of the animal ethics committee of the university of medical sciences.

All methods, including anesthesia and sacrifice procedure were conducted in accordance with the guide for the care and use of laboratory animals, institutes for laboratory animal research, National Institutes of Health (NIH Publication No.85–23, revised 1996) and approved by the Animal Ethics Committee of University of Medical Sciences (IR.IUMS.REC1395.28895).

### Exercise protocols

In order to familiarize with treadmills, rats in exercise training groups practiced 3 sessions at a speed of 20 m/min for 10 to 15 min (approximately 50% of VO2max) [21, 22]. Then, after 1 day rest, the two-week protocol was performed in the same way as Table 1, which included four sections. The first section consisted 3 days of training, two sessions each day, and each session consisting of 4 × 2 min with the speed of 35–40 m/min (~ 85–85% of vo2max) and 3 × 2 min with slow speeds of 25 to 30 m /min (~ 60–60% vo2max) between two high interval training. The second part included 2 days of training, 4 × 2 min with 40 to 45 m / min (~ 95% -0 to 50% vo2max) and 3 × 2 min slow intervals with 28 to 32 m /min (~ 75% vo2max75). The third part included 3 days of training, including 5 high intervals and 4 slow intervals, and the intensity of this section was similar to the second part. The fourth section consists of 2 days of training, as in the third part, but with 6 high intensity intervals and 5 slow intensity intervals [23].

**Table 1:** Training protocol

	First week		Second week	
	First section	Second section	Third section	Fourth section
	3 days 2 sessions each day	2 days 2 sessions each day	3 days 2 sessions each day	2 days 2 sessions each day
Maximum speed (m/min)	High: 37 Slow: 26	High: 42 Slow: 33	High: 42 Slow:33	High: 42 Slow: 33
Duration (min)	14	14	18	22
Intervals	High: 4 sets Slow: 3 sets	High: 4 sets Slow: 3 sets	High: 5 sets Slow: 4 sets	High: 6 sets Slow: 5 sets
Running distance per session (m)	452	528	684	834
Running distance per section (m)	2712	2112	4104	3336

### Endurance capacity

**Fig. 1:**
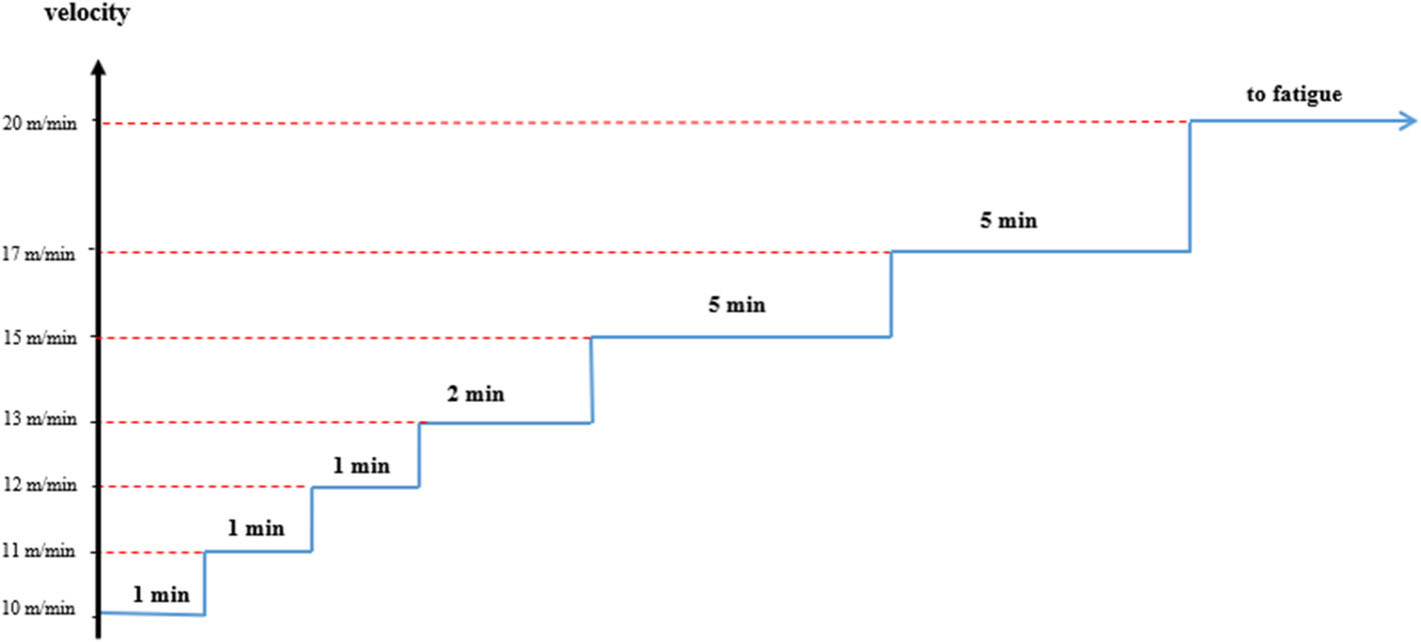
The steps of the graded and exhaustion exercise test in order to evaluate of endurance capacity

In order to ensure the effectiveness of exercise training and measurement of endurance capacity (the highest rate of oxygen consumption attainable during maximal or exhaustive exercise), the maximum exercise performance test was performed at the beginning and the end of the exercise training. The protocol consisted of warm up with intensity of 15 to 25 m/min for 5 min and performance test with the speed and time as shown in Fig. 1 [23]. Exercise performance was determined from a graded exercise test to exhaustion performed at 0% incline, beginning with 10 m/min for 1 min, 11 m/min for 1 min, 12 m/min for 1 min, 13 m/min for 2 min, 15 m/min for 5 min, 17 m/min for 5 min and 20 m/min until exhaustion. Exercise performance was determined by the time to exhaustion. Twice hit with the shock device (electrical stimulus 0.5 mA at the end of the treadmill) at the end of the treadmill in 30 s or reflection of the return and standing upright on the leg were considered as exhaustion [24].

### Induction of myocardial ischemia

For induction of myocardial infarction, subcutaneous injection of Isoprenaline (150 mg/kg) solution in normal salin was performed for two consecutive days (24 h after the first injection, the second injection was performed). The use of this substance in animal models, especially in rats, is one of the common ways to cause infarction [25].

### Confirmation of infarction

Blood enzymes creatine kinase (CK), lactate dehydrogenase (LDH), and troponin T were measured to confirm the injury of the heart tissue. Hematoxylin and Eosin (H&E) staining was also used to investigate the necrosis and Mason’s trichrome staining was used to examine the fibrous tissue.

### Protein measurement

The G-CSF and G-CSFR (CD114) levels were measured using the Western Blot method. One hundred milligram left ventricular tissue was Lysed and homogenized in the Radioimmunoprecipitation assay buffer (RIPA buffer) for 30 min, then centrifuged at a temperature of 4 °C at 12,000 rpm for 20 min and the supernatant was separated. Nanodrop evaluation was used to determine the protein concentration. Protein samples were combined equally with a sample buffer (10 ml Tris (PH = 6.8), 12.5 ml Glycerol, 2.5 ml β -mercapto ethanol, 0.01 g Bromo phenol Blue, 25 ml SDS 10%)) and boiled at 100 °C for 7 min. The solution was then exposed to sodium dodecyl sulfate–polyacrylamide gel electrophoresis (SDS-page) 12.5% and the proteins were transferred to the Polyvinylidene difluoride membrane (PVDF membrane). Subsequently, the amounts of these proteins were identified by primary antibodies of G-CSF (England, Biorbyt, orb10702) and G-CSFR (England, Biorbyt, orb13429). Finally, the membrane was incubated with an ECL Western blotting system and exposed to X-ray film. Band density analysis was performed by image j software. Since β-actin is a component of proteins whose expression is constant in the cell [26], the antibody of this protein was used to eliminate the error of loading the equal amounts of protein in the wells.

### Statistical analysis

In the present study, the Shapiro-Wilk test was used to determine the normal distribution of data and one-way ANOVA and Tukey tests were used for data analysis. Statistical analysis was performed using SPSS software. All values were presented as means ± SD and *P* < 0.05 was considered statistically significant.

## Results

### Serum markers of cellular injury and confirmation of necrosis and fibrous

**Fig. 2:**
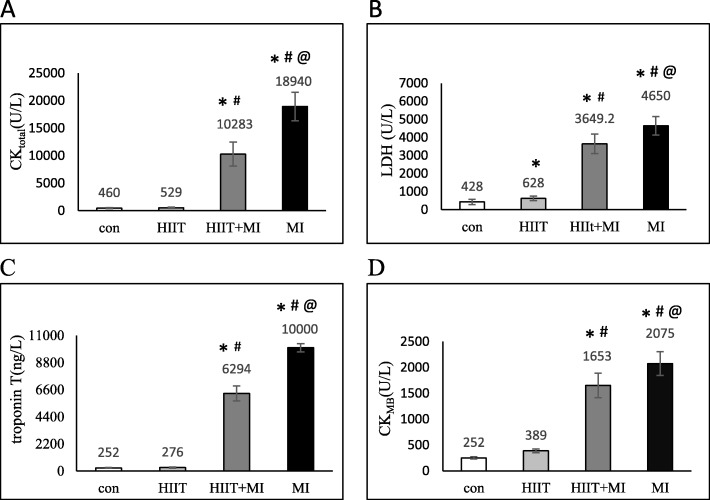
Cell injury markers in experimental groups. Results were presented as means ± SD. **a**: Blood levels of creatine kinase total (CK_total_ ), **b**: blood levels of lactate dehydrogenase (LDH), **c**: Blood levels of troponin T (cTI), **d**: Blood levels of creatine kinase (CK.). * *p* < 0.001 compared to the control; # *p* < 0.05 compared to the HIIT, and @ *p* < 0.001 compared to the HIIT+MI

As shown in Fig. 2, the results of this study showed a significant increase in LDH, CKMB, CK total and troponin-T following 2 weeks of HIIT and infarction (*P* < 0.001). Tukey’s post hoc test showed that induction of infarction significantly increased these factors in the MI group and HIIT+MI group compared to control and HIIT groups (*P* < 0.001). Also, the results showed that the exercise training does not increase these cell injury indices in the HIIT+MI group than in the MI group, which indicates the cardioprotection effect of the exercise training. There is also a significant difference in the LDH enzyme between the training group and the control group (*p* = 0.042).

**Fig. 3:**
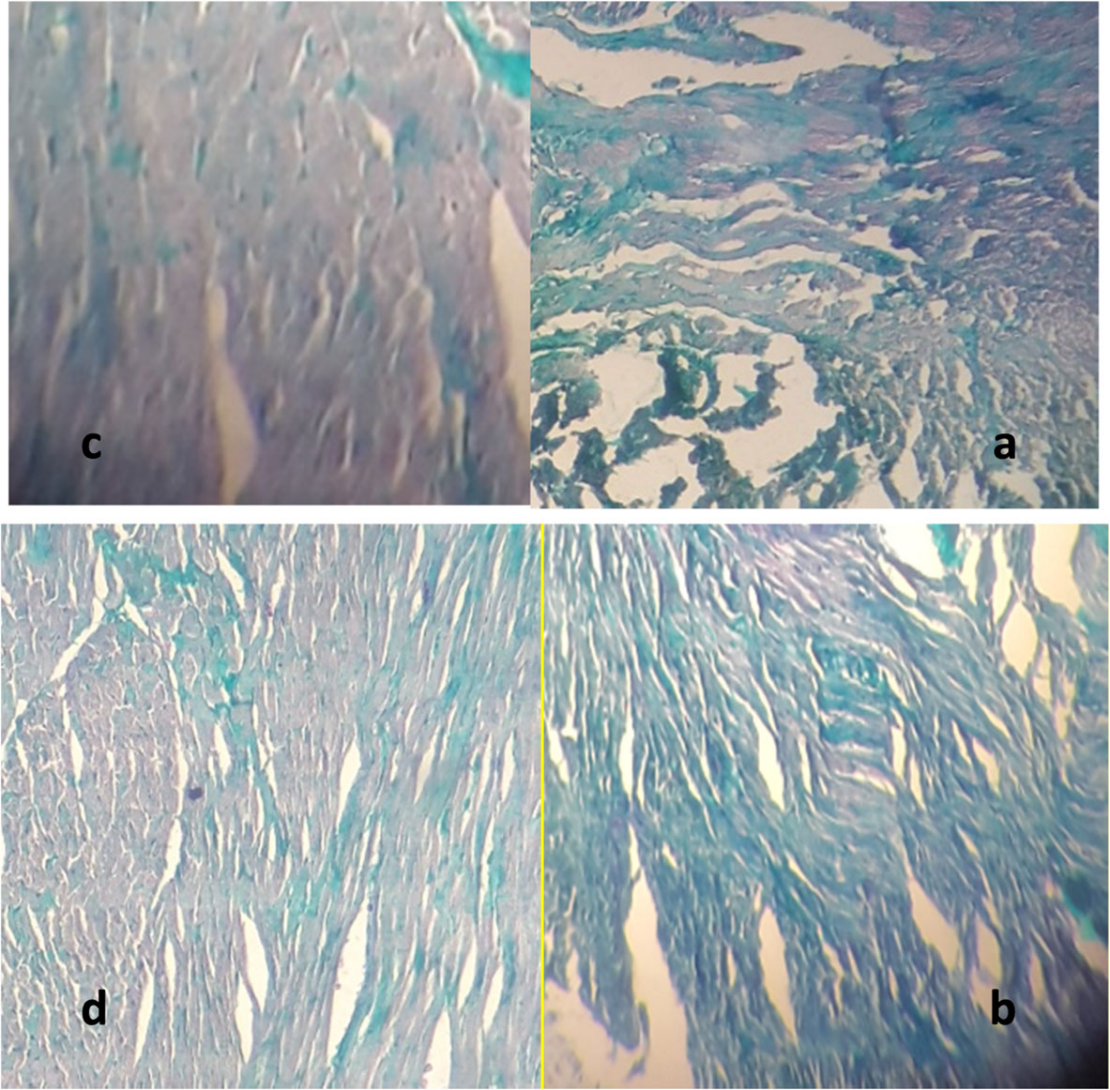
The amount of fibrosis in the heart tissue in (**a**) control group, **b** HIIT group, **c** MI group, and (**d**) HIIT+MI group. The green color represents the amount of tissue fibrosis (× 40 magnification)

**Fig. 4:**
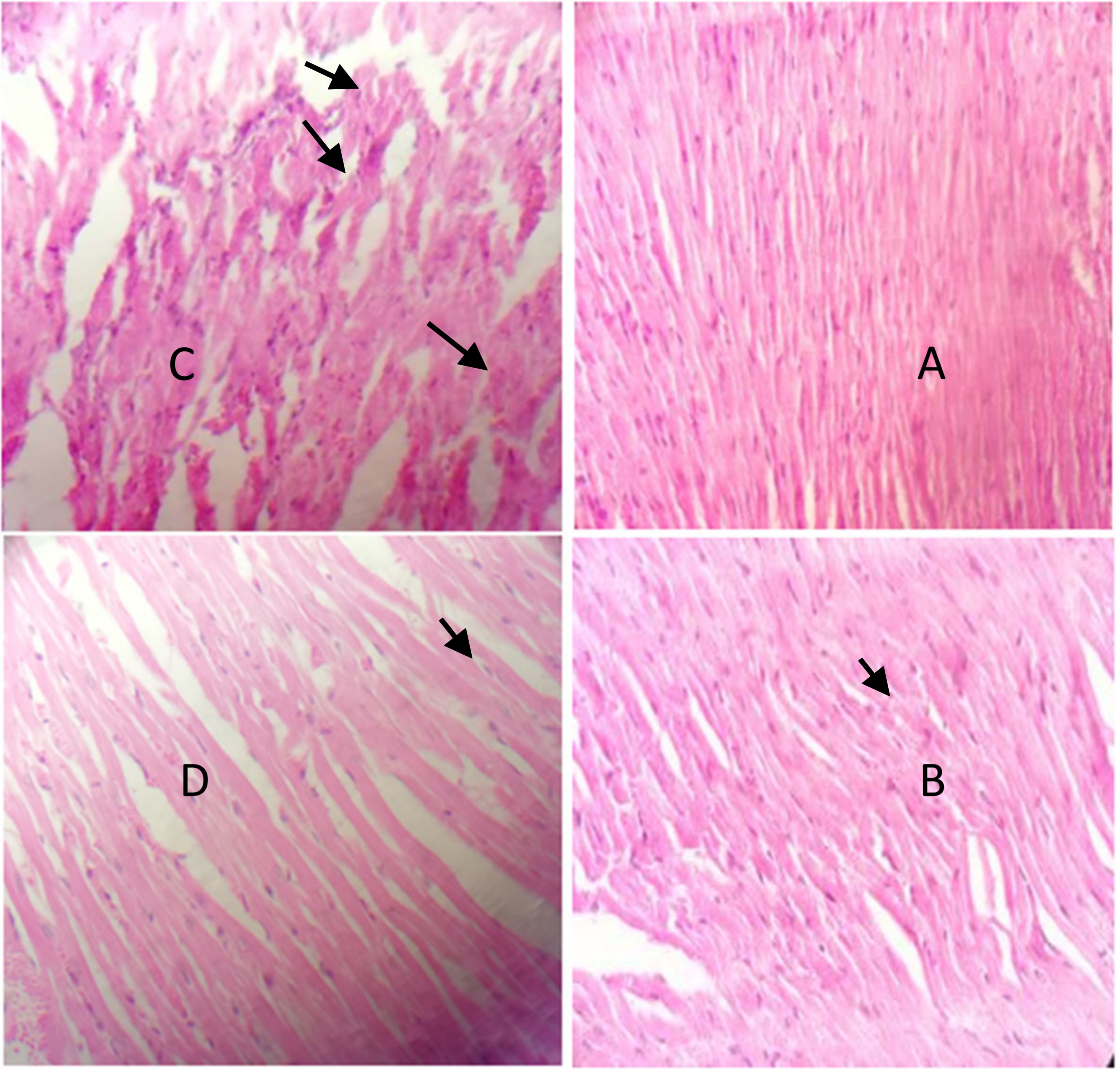
Necrosis in the heart tissue in (**a**) control, **b** HIIT, **c** MI, and **d** HIIT+MI groups. Neutrophil accumulation, edema and separation of fibers (Arrows show) (× 40 magnification)

Mason’s trichrome staining (Fig. 3) and Hematoxylin and Eosin (H&E) staining results (Fig. 4) was shown the amount of fibrosis and necrosis in the heart tissue and can confirm the protective effect of exercise training against infarction.

### Protein expression

**Fig. 5:**
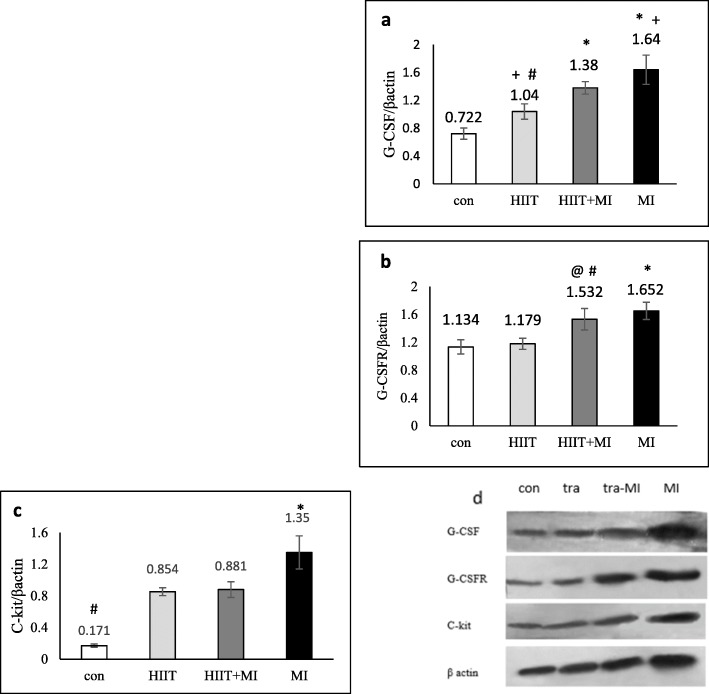
Protein expression in experimental groups (**a**, **b**, **c**, **d**). **a**: * *p* < 0.001 and # *p* < 0.05 compared to the control group; + *p* < 0.05 compared to the HIIT+MI. **b**: * *p* < 0.001 compared to the control and HIIT groups; # *p* < 0.01 compared to the control group; + *p* < 0.05 compared to the HIIT group. **c**: * *p* < 0.001 compared to 3 other groups; # *p* < 0.001compared to 3 other groups. **d**: Western blot images and comparison of the protein bands of G-CSF, G-CSFR, C-kit with β-actin (as a loading control) between groups. The ratio of these proteins to β-actin expression in experimental groups was shown and described in a, b, c sections

The results of western blot analysis showed that the G-CSF protein had a significant increase after 2 weeks of HIIT and MI (Fig. 5a). Tukey’s post hoc test showed that this protein in the MI group had a significant increase compared to the HIIT (*P* < 0.001), HIIT+MI (*P* = 0.042) and control (*P* < 0.001) groups. Moreover, there was a significant increase in the HIIT (*P* = 0.019) and HIIT+MI (*P* < 0.001) groups compared to the control group. Also, there was a significant difference between HIIT+MI and HIIT (*P* = 0.016).

The expression of the G-CSFR was also assessed in four groups using the western blot technique. The result in Fig. 5b shows that the amount of this protein in the MI group has a significant increase compared to the control (*P* < 0.001) and HIIT (*P* < 0.001) groups. Also HIIT+MI group has a significant increase compared to the control (*P* = 0.001) and HIIT (*P* = 0.004) groups.

The value of C-kit protein in the HIIT and HIIT+MI groups showed a significant increase compared to the control group (*P* < 0.001). Quantitative results are also seen in Fig. 5c. Also, the C-Kit protein content in the MI group was significantly higher than other groups (*P* < 0.001).

### Endurance capacity

The results of this study showed that 2 weeks of High-intensity interval training significantly increased (*P* < 0.001) endurance capacity in the two trained groups, which represents the effect of this duration and type of exercise on the endurance capacity in rats. The running distance in the control group was 735 m and the time was 41.5 min at the beginning of the training, which after 2 weeks was 945 m and its time to 48.95 min. Also, the running distance in the MI group changed from 774 m to 976 m and its time changed from 42.75 to 52.85, but the running distance in the training group at the beginning of the training was 760 m and the time was 41.86 min, but after 2 weeks of training, the distance changed to 3400 m and the time to 182.75 min. In the HIIT+MI group, running distance changed from 723 to 3324 m and the time from 40.2 to 170.25 min. These results are summarized in Table 2.

**Table 2:** Running distance and time spent before and after 2 weeks of HIIT in four research groups

Groups	Parameters	Before Training	After Training
Control	Running distance (m)	735	945
	Time spent (m)	41.05	48.95
MI	Running distance (m)	774	976
	Time spent (m)	42.75	52.85
HIIT	Running distance (m)	760	3400
	Time spent (m)	41.86	182.75
HIIT+MI	Running distance (m)	723	3324
	Time spent (m)	40.2	170.25

## Discussion

Pathological results of the present study showed that injection of isoprenaline causes severe tissue injury to the heart. As shown in Fig. 4, in the MI group, severe pathological changes were created, including edema, neutrophil accumulation, separation of fibers, hemorrhage, and collagen formation in the heart tissue. These findings were similar to the results of Farvin et al. (2010) and Tofighi et al. (2016) studies [27, 28]. LDH, CKMB, CKtotal and troponin-T levels in the MI group increased more than the other three groups, indicating a higher degree of injury in this group. The results of this study were consistent with some studies [29–31]. In the present study, it was shown that the level of G-CSF protein and its receptor) G-CSFR) and C-kit in the heart tissue significantly increased in the MI group. Under normal conditions, the amount of stem cells and stem cell mobilizations factors are negligible, but it has been reported that their amounts or activity gets high immediately after ischemia and heart damage, as a body’s inherent protective mechanism [32]. Increasing these factors improves left ventricular function after injury. Possible mechanisms of this event can be attributed to the repair of the heart tissue by the process of vasculogenesis and angiogenesis [33], reduction of cardiac remodeling [34], and triggering of paracrine mechanisms for repairing damaged myocytes and antiapoptotic and antifibrotic signalings [6, 33].

Stem cells and factors requirement of these cells such as G-CSF, SDF, C-kit is one of the signaling pathways of exercise-induced cardiac hypertrophy. The levels of G-CSF and its receptor in various tissues are negligible [8], but the concentration of these factors changes in different physiological conditions, such as ischemia and exercise- induced hypoxia [10, 11]. Baker et al. showed a significant increase in skeletal muscle G-CSF and stem cell after endurance training [14]. The results of Baker et al. study are similar to the present study, which investigated the effect of HIIT on tissue G-CSF concentrations. C-kit in the heart tissue significantly increased in the MI group versus other groups. Increasing the blood level of G-CSF after exercise training was shown in some studies, which can increase the release and mobilization of stem cells from bone marrow and ultimately their migration to the heart, and the differentiation of these cells and increase the physiologic hypertrophy of the heart [35–38].

Another important effect of exercise training is the increase of the protective ability of various tissues, against probable injuries, such as heart infarction. As the results of this study showed, the level of heart injury indices (LDH، CKMB، CKtotal, troponinT) in the HIIT+IR group was lower than IR group. Also, the level of stem cell recruitment factors that increased during tissue injury, was lower in the HIIT+IR group than the IR group that this also indicates a lower level of injury in the HIIT+IR group. H & E staining results can also confirm the protective effect of exercise training against infarction. The results of this study were similar to some studies, which showed that exercise training increased the tissue protective ability through various mechanisms [29–31]. Regular exercise training can increase cardioprotection through anatomical and physiological changes in the coronary arteries, and cellular-molecular mechanisms such as induction of myocardial shock shock (HSPS), increased activity of cyclooxygenase-2 (COX-2), increased endoplasmic endothelial stress (ER), increased levels of ATP-dependent potassium in sarcoloma (sarcoKATP) and mitochondria (mitoKATP), nitric oxide (NO), and increased cardiac antioxidant capacity [39, 40].

The increase in G-CSF induced by physical activity in addition to mobilize stem cell types in different tissues of the body can activate other mechanisms such as inhibition of apoptotic pathways, increased angiogenesis, endothelial regeneration*,* proliferation and differentiation of the heart cells. The binding of G-CSF to its receptors (G-CSFR) activates the JAK/STAT signaling pathways, Akt and BCL2 and inhibits BAX and caspase-3, and affects the BAX/BCL2 ratio (cell death index), and protects from myocytes by reducing and inhibiting apoptosis [41]. It was reported that the AMI-induced scar tissue area after G-CSF therapy was significantly reduced [42]. Both exercise training and infarction significantly increase the C-kit protein in the heart tissue. The results of this study are similar to the results of the study by Klein et al., which have shown that ischemia in the brain tissue increases the expression of the C-kit [43]. C-kit is a kinase receptor that is required for the proliferation, survival and migration of stem cell types [44] and is recognized as one of the stem cell markers [43]. It has been shown that increasing the expression of the C-kit facilitates the migration of stem cells to the damaged heart tissue by activating the P38 MAPK [45]. It has also been shown that these receptors can protect the heart against various injuries by activating the pathway of Akt and ERK1/2, and even contribute to the repair of the damaged area [46]. Also, a number of studies have reported that changes in the levels of G-CSF and stem cells are not induced as a result of exercise training [21, 22, 47]. The reason for not changing these factors in these studies is probably related to the type and intensity of the exercise training protocols. Because the exercise protocols used in the some of them included self-made and habitual movements [21], some of them were mild walking on treadmill [22] or weightless exercise [10]. Conversely, in researches that show the increase of progenitor cells, has been used high intensity exercise training protocols. Therefore, increasing the level of G-CSF and C-kit in tissues through any physiological agent (exercise, hypoxia,...) can promote cell and tissue growth or protect the tissue against damage.

## Conclusions

Regular physical activity can protect the heart against different stresses through various approaches that one of them, which today is paying a lot of attention, is increasing the level of stem cells and stem cell mobilization factors. The results of this study showed that exercise training can reduce cardiac damage caused by acute myocardial infarction and protect the heart by increasing stem cell recruitment factors.

## Data Availability

The datasets used and/or analysed in the current study are available from the corresponding author upon reasonable request.

## Abbreviations

ATP: Adenosine triphosphate
CK: Creatin kinase
Flt3: FMS-like tyrosine kinase 3
G-CSF: Granulocyte colony-stimulating factor
H&E: Hematoxylin and eosin staining
HIIT: High intensity interval training
LDH: Lactate dehydrogenase
PVDF: Polyvinylidene difluoride membrane
RIPA: Radioimmunoprecipitation assay buffer
RNS: Reactive nitrogen species
ROS: Reactive oxygen species
SCF: Stem cell factor
SDF: Stromal cell-derived factor
SDS-page: Sodium dodecyl sulfate–polyacrylamide gel electrophoresis
